# Trend in the incidence of commuting accidents among workers in Brazil between 2009 and 2016

**DOI:** 10.5327/Z1679443520190439

**Published:** 2019-12-01

**Authors:** Arthur Arantes Cunha, Rodolfo Antonio Corona, Danielle Gonçalves Silva, Amanda Alves Fecury, Claudio Alberto Gellis de Mattos Dias, Maria Helena Mendonça Araújo

**Affiliations:** 1 Undergraduate medical course, Universidade Federal do Amapá - Macapá (AP), Brazil. Universidade Federal do Amapá Undergraduate medical course Universidade Federal do Amapá Brazil; 2 Undegraduate law course, Universidade Federal do Amapá - Macapá (AP), Brazil. Universidade Federal do Amapá Undegraduate law course Universidade Federal do Amapá Brazil; 3 Undergraduate chemistry course, Universidade Federal do Amapá - Macapá (AP), Brazil. Universidade Federal do Amapá Undergraduate chemistry course Universidade Federal do Amapá Brazil

**Keywords:** occupational health, accidents, occupational, occupational medicine, social security

## Abstract

**Background:**

Commuting accidents might have serious consequences for the health of workers, in addition to considerable financial impacts on the national health system and the social security administration.

**Objective:**

To describe the epidemiological profile and calculate the incidence of commuting accidents in Brazil in the period from 2009 to 2016.

**Methods:**

Retrospective and descriptive study with time series analysis (2009-2016) based on official secondary data obtained from Social Security Statistical Yearbooks. Information on the economically active population was retrieved from the National Household Sample Survey. Annual incidence rates were calculated per 100,000 economically active population. Simple temporal linear regression analysis was performed with software Statistical Package for the Social Sciences. The significance level was set to p<0.05.

**Results:**

The epidemiological profile of workers involved in commuting accidents corresponded to men, aged 20 to 29 and with leg/ankle fractures, which represented 24.78% of the total population of involved workers. The incidence of commuting accidents increased from 88.17 to 105.88 in the analyzed period, which represents an variation rate of 20.08% (R^2^=0.715; p=0.008).

**Conclusion:**

The detected rise in the incidence and number of commuting accidents point to the need for the government to formulate prevention plans targeting high-risk groups.

## RESUMO |

**Introdução:**

Os acidentes de trajeto ocorrem no percurso do domicílio até o local de trabalho e vice-versa. Esse tipo de acidente de trabalho pode resultar em grave desordem à saúde do trabalhador e, em grande quantidade, impactar financeiramente o Sistema Único de Saúde e a Previdência Social Brasileira (PSB).

**Objetivos:**

Descrever o perfil epidemiológico e estimar a incidência dos acidentes de trajeto em trabalhadores no Brasil entre 2009 e 2016.

**Métodos:**

Estudo retrospectivo descritivo, com análises de séries temporais (2009-2016), baseado em dados secundários e oficiais extraídos dos Anuários Estatísticos da Previdência Social. As informações de População Economicamente Ativa foram retiradas da Pesquisa Nacional por Amostra de Domicílios. As incidências anuais foram calculadas para cada 100 mil pessoas economicamente ativas, independentemente de sexo, de idade e de contribuição para a PSB. Utilizou-se o Statistical Package for the Social Sciences (SPSS) para realizar testes de regressão linear simples temporal, considerando significância de p<0,05.

**Resultados:**

O perfil epidemiológico do trabalhador acometido foi de homem entre 20 e 29 anos com fratura de perna e/ou tornozelo, que representou 24,73% do total de trabalhadores acometidos. No período, a incidência dos acidentes de trajeto aumentou de 88,17 para 105,88, o que resultou em taxa de variação de 20,08% (R^2^=0,715; p=0,008).

**Conclusão:**

O crescimento da incidência e do número de acidentes demonstra grande necessidade de elaboração de planos governamentais de prevenção, que devem ser direcionados aos principais grupos de risco.

## INTRODUCTION

The Law no. 8213, on the rights of workers involved in accidents outside the workplace and working hours, was passed in Brazil in 1991^1^. This piece of legislation contributed to reorganize the labor rights and also anticipated and characterized the Work Accident Report (WAR) forms currently in force, which require reporting the type of incidents^2^. One such types are commuting accidents, which are intrinsically related to urban modes of transport used by Brazilian workers, such as cars, buses and bicycles, among others, but also include pedestrians^3^^,^^4^. It should be observed that the legislation on commuting accidents goes much beyond reports and numbers, because it can be also seen as a true policy of stability for workers, therefore, a significant labor right^2^.

Commuting accidents are incidents which occur on the way between home and the workplace in either direction^1^^,^^3^. Their comprehension also includes the workers with jobs related to urban traffic, such as truck and taxi drivers while on the routes alluded in the law^1^^,^^4^. These incidents may result in body injuries or functional disorders liable to cause permanent or temporary loss or reduction of the capacity for work, and even death^1^. Two other types of work accidents are the typical and those associated with work-related diseases^5^.

The vast majority of commuting accidents take place in the urban environment and are associated considerable hospital and social security costs. Sex and age have been described as possible risk factors^3^^,^^6^. The overall rate of accidents among workers covered by the Brazilian Social Security system decreased by 17.04% from 1998 to 2008. However, the rate of commuting accidents increased from 1.7 to 2.4/1,000 workers, i.e. 41.18%, in the same period. The number of commuting accidents more than doubled, having risen from 26,016 in 1998 to 66,217 in 2008^7^. According to Bin^8^, this trend of increase is global, especially as concerns developing countries.

Commuting accidents are intrinsically related to vehicle traffic, urban growth and violence and have strong financial impacts for the social security system in terms of sick leaves, disability retirement and death, and also for the national health system in relation to hospitalization and surgical procedures^6^^,^^9^. About 7,000 deaths occurred in Brazil from 2006 to 2008 as a result of work accidents, considering only workers covered by the social security system^10^. These data point to the relevance of studying this type of incidents having health of workers as the main focus of concern beyond the considerable economic cost^3^^,^^6^.

Epidemiological information on the characteristics of victims of commuting accidents and resulting injuries allows identifying personal risk factors, the cost of treatments and loss of productivity^3^^,^^9^. Commuting accidents have mainly impact on the economically active population, the national health system, the social security administration and the national economy. For this reason, establishing the characteristics of the involved workers has paramount importance.^3^^,^^11^

Given the impact of commuting accidents on the Brazilian society and the still small number of studies on this subject, the aims of the present study were to describe the epidemiological profile (sex, age and injuries) of workers involved in commuting accidents, perform time series analysis of their incidence and calculate their rate relative to the total number of work accidents for which WAR were issued in the period from 2009 to 2016.

## METHODS

### DATA SOURCES AND VARIABLES

The present retrospective and descriptive study, including time series analysis (2009-2016), was based on open-access, secondary and official data collected and made available by the Brazilian social security administration and the Brazilian Institute of Geography and Statistics (IBGE). Information on accidents was obtained from the Social Security Statistical Yearbooks for 2016^5^, 2014^12^, 2012^13^ and 2010^14^. Each such yearbook provides data for the year of publication and the two preceding ones.

We considered only data relative to work accidents for which WAR were registered by the National Social Security Institute (INSS). The reason is that while duly reported and quantitatively recorded, incidents not registered by INSS lack information on their type (the three aforementioned ones, as defined by the social security administration). Data for incidents registered by INSS also include the age and sex of victims and describe injuries according to the 10th revision of the International Classification of Diseases (ICD-10).

Data on the economically active population necessary to calculate the annual incidence of commuting accidents in the period from 2009 to 2016 were obtained from IBGE Continuous National Household Sample Survey (Continuous PNAD)^15^^,^^16^. The economically active population includes both employed and unemployed individuals independently of whether or not they contribute to the social security system. The annual incidence of commuting accidents (number of commuting accidents x 100,000/economically active population) was calculated independently from the age and sex of the involved workers, because this information is not provided by Continuous PNAD^15^^,^^16^.

We analyzed open-access aggregate data in the public domain as per the Law no. 12,527/2011^17^. The present study complies with the National Health Council Resolutions no. 466/2012^18^ and 510/2016^19^.

### DATA ANALYSIS

The data were processed, tabulated and represented graphically using software Microsoft Excel 2016 and Statistical Package for the Social Sciences (SPSS) version 20.0. We performed simple temporal linear regression analysis of the incidence of communing accidents, number of commuting accidents according to sex, rate of commuting accidents relative to the total number of work accidents with duly registered WAR and the quantitative distribution of the three most frequent types of injuries. We fitted seven linear regression models, one for each year considered. The linear models were fitted for each analyzed variable, Y=ß_0_+ß_1_X, in which X corresponds to the analyzed year and Y to the quantitative variable under analysis^20^^,^^21^. We calculated coefficients of determination (R^2^) and detected trends of growth or reduction.

Through the F test for analysis of variance we calculated p values and estimated the goodness-of-fit of each model. The significance level for this test was set to p<0.05. Finally, we calculated mean and standard deviation (SD) for each analyzed variable^20^^,^^21^.

## RESULTS

**Figure 1.**
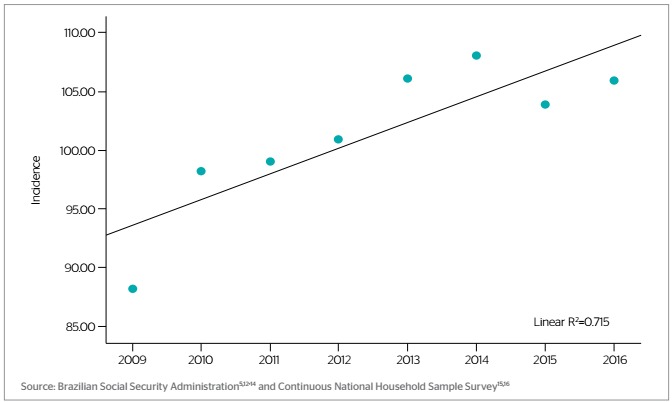
Incidence of commuting accidents, Brazil, 2009-2016

Source: Brazilian Social Security Administration^5^^,^^12^^,^^13^^,^^14^ and Continuous National Household Sample Survey^15^^,^^16^

The incidence of commuting accidents increased from 88.17 in 2009 to 105.88 in 105.88, i.e. by 20.08%. The highest incidence corresponded to 2014 and the lowest to 2009; mean incidence was 101.28, SD=6.37. The angular coefficient of the regression model (Y=2.1615X-4,248.83; R^2^=0.715; p=0.008) indicates that incidence increased by 2.16 cases/100,000 economically active population/year, on average (Figure 1).

On separate analysis of the three types of work accidents considered, only the relative rate of commuting accidents increased. While in 2009 they represented 16.88% of incidents, in 2016 this rate increased to 22.78%, with significant percent variance of 34.95%. The highest relative rate in the analyzed period corresponded to 2016 and the lowest to 2009. The angular coefficient of the regression model (Y=0.7564X-1,500.7; R^2^=0.969; p<0.001) indicates that the relative rate increased 0.75%/year (Table 1).

**Table 1.** Progression of commuting, typical and accidents due to work related diseases, Brazil, 2009-2016

Year	Typical accidents		Commuting accidents		Accidents due to work-related diseases		Total
	No. cases	%	No. cases	%	No. cases	%	No. cases
2009	424,498	79.46	90,180	16.88	19,570	3.66	534,248
2010	417,295	78.77	95,321	17.99	17,177	3.24	529,793
2011	426,153	78.35	100,897	18.55	16,839	3.1	543,889
2012	426,284	78.04	103,039	18.86	16,898	3.09	546,222
2013	434,339	77.05	112,182	19.9	17,182	3.05	563,704
2014	430,454	76.28	116,228	20.6	17,599	3.12	564,283
2015	385,646	75.95	106,721	21.02	15,386	3.03	507,753
2016	354,084	74.59	108,150	22.78	12,502	2.63	474,736
Mean	412,344.12	----	104,089.75	----	16,644.12	----	----
Standard deviation	27,969.39	----	8,601,.9	----	2,031.47	----	----
Total	3,298,753	----	832,718	----	133,153	----	----

Fonte: Brazilian Social Security Administration^5^^,^^12^^,^^13^^,^^14^

Most victims of commuting accidents were male, n=519,828 (64.42%) (mean: 64,978.50, SD=4,026.03) versus women n=312,890 (37.58%) (mean: 39,111.25, SD=5,024.32). The total number of incidents was 832,718, with a difference of 206,938 between both sexes. Percent variance of the number of accidents in the analyzed period was higher for the women (+39.36%) compared to the men (+9.58%). For both sexes the largest number of incidents occurred in 2014, men n=71,458, women n=44,770 (Table 2).

**Table 2.** Commuting accidents per age and sex, Brazil, 2009-2016

	2009	2010	2011	2012	2013	2014	2015	2016	%	Total
Males										
≤ 19 years old	2,424	2,670	2,859	2,827	3,000	3,014	2,435	2,139	4.1%	21,368
20-29 years old	25,268	26,206	26,870	25,857	27,162	27,200	23,724	23,651	39.6%	205,938
30-39 years old	16,830	17,819	18,624	19,550	21,477	22,368	20,332	20,740	30.4%	157,740
40-49 years old	9,475	10,031	10,410	10,508	11,429	11,826	10,840	11,096	16.5%	85,615
50-59 years old	4,068	4,312	4,670	4,857	5,441	5,685	5,504	5,496	7.7%	40,033
60-69 years old	734	809	936	1,024	1,190	1,262	1,155	1,275	1.6%	8,385
≥ 70 years old	47	49	93	68	107	98	100	101	0.1%	663
Total	58,846	61,896	64,462	64,691	69,806	71,453	64,090	64,498	100.0%	519,742
Females										
≤ 19 years old	1,016	1,110	1,184	1,204	1,501	1,478	1,308	1,127	3.2%	9,928
20-29 years old	11,835	12,631	13,323	13,452	14,873	15,528	14,168	14,303	35.2%	110,113
30-39 years old	9,023	9,744	11,080	11,909	13,420	13,998	13,688	14,121	31.0%	96,983
40-49 years old	5,832	6,091	6,685	7,209	7,468	8,262	7,864	8,227	18.4%	57,638
50-59 years old	3,100	3,367	3,616	3,913	4,413	4,680	4,708	4,878	10.5%	32,675
60-69 years old	390	429	491	585	654	765	839	932	1.6%	5,085
≥ 70 years old	22	36	41	22	48	59	54	63	0.1%	345
Total	31,218	33,408	36,420	38,294	42,377	44,770	42,629	43,651	100.0%	312,767

Brazilian Social Security Administration^5^^,^^12^^,^^13^^,^^14^

The model fitted for the number of incidents involving males (Y=909.85X-1,766,109.0; R^2^=0.306; p=0.154) indicated a trend of increase, however, it was not statistically significant. In turn, the results of the model fitted for the women (Y=1,922.6X-3,830,111.6; R^2^=0.878; p<0.001) exhibited a statistically significant increasing trend; the angular coefficient indicates that the number of incidents increased by 1,922.6/year, on average.

For the men, the highest number of accidents corresponded to age range 20 to 29, n=205,938, representing 39.62% of the total of incidents involving male victims. This group was followed by that aged 30 to 39, n=157,740, 30.35%. For the women, the highest number of accidents also corresponded to age range 20 to 29, n=110,113, representing 35.20% of the total of incidents involving female victims. This group was followed by that aged 30 to 39, n=96,983, 31.00% (Table 2).

**Figure 2.**
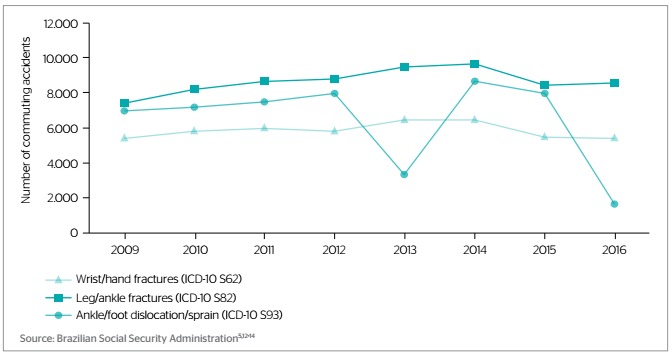
Most frequent injuries associated with commuting accidents as per the 10th revision of the International Classification of Diseases (ICD-10), Brazil, 2009-2016

Source: Brazilian Social Security Administration^5^^,^^12^^,^^13^^,^^14^

The three most frequent types of injury following commuting accidents as per ICD-10 code were fracture of the lower leg, including ankle (ICD-10 S82), n=69,480 (mean: 8,085.00, SD=713.07), dislocation and sprain of joints and ligaments at ankle, foot and toe level (ICD-10 S93), n=51,470 (mean: 6,433.75, SD=2,524.82) and fracture at wrist and hand level (ICD-10 S62), n=47,444 (mean: 5,930.50, SD=424.32). Together they correspond to 168,394 incidents and 20.22% of the total of commuting accidents in the analyzed period (Figure 2).

The regression model fitted for joint and ligament dislocation and sprain (ICD-10 S93) evidenced a statistically nonsignificant decreasing trend (Y=-410.76X+833,092.0; R^2^=0.159; p=0.328). The models fitted for leg fractures (ICD-10 S82) and wrist/hand fractures (ICD-10 S62) evidenced statistically nonsignificant increasing trends (Y=155.81X-304,881.6; R^2^=0.286; p=0.171 and Y=2.9286X+36.7528; R^2^=0.001; p=0.968, respectively).

Thus we were able to characterize the epidemiological profile of workers involved in commuting accidents as male, aged 20 to 29 and resulting in lower limb/ankle fractures (ICD-10 S82) which represented 24.73% of the total number of incidents registered in the period from 2009 to 2016.

## DISCUSSION

The results of regression analysis indicate an increase of 20.08% in the incidence of commuting accidents in the period from 2009 to 2016. A similar finding was reported by Pinto^22^ for Brazil from 2008 to 2013 based on moving average analysis and the Hodrick-Prescott filter. The results indicated relative stability of the incidence of accidents in the analyzed period, with a trend to increase starting in 2012 as shown by the moving average method. Differently, in Almeida et al.’s study^10^, who analyzed the period from 1998 to 2008 by means of joinpoint regression analysis, the results indicated a clear increasing trend in the incidence of commuting accidents. 

Joint analysis of Almeida et al.’s^10^ and our results, despite the differences in the analyzed populations, shows that the incidence of commuting accidents exhibits a trend of increase since 1998. This increase is explained by a larger number of incidents, since according to Continuous PNAD^15^^,^^16^ the economically active population increased in the period from 2009 to 2016. This rise in the number of commuting accidents might be due to the growth of urban violence, which pendular motion affects ever more and more workers 7.

Another reason for the growth in the number of commuting accidents might be the increase in the rate of motor vehicles/population, in turn associated with higher rates of traffic accidents^7^. Other factors to take into account are lack of awareness on safe driving among workers and the scarcity of commuting safety management programs. These factors, which influence the occurrence of traffic accidents, might also be associated with commuting accidents, since according to the International Labor Organization both types of incidents are closely interrelated^8^.

Our results evidence an increasing trend in the frequency of commuting accidents relative to the total of work accidents for which WAR were registered. This rate rose from 16.88% to 22.78%, i.e. 34.95%. Our findings corroborate those reported by Wünsch Filho^23^, who performed nationwide analysis of time intervals within the period from 1980 to 2000 and found a progressive rise from 3.80% to 10.90% in the rate of participation of commuting accidents. According to this author, the rates of commuting accidents and those associated with work-related diseases increased, while that of typical accidents decreased. In the present study we found increasing participation of commuting accidents and decrease of the other two types. Joint analysis of these data point to a rise in the relative proportion of commuting accidents in the period from 1980 to 2016 relative to the total number of work accidents with duly registered WAR.

This rise in the participation of commuting accidents should not only be attributed to an elevation of their absolute number, but also to a decrease in the occurrence of typical accidents resulting from increasing underreporting-particularly in the case of outsourced employees-and higher investment in workplace health and safety. However, few studies analyzed the actual effects of such investment on the occurrence of accidents^7^^,^^9^.

Commuting accidents have significant impacts on the health of workers, leading to loss of productivity and discontinuation of occupational activities, in addition to the costs of medical care^9^. Within this context, the results of the present study indicate that limb fractures (ICD-10 S62 and S82) are very frequent outcomes of commuting accidents. This type of injuries is very common following land transport accidents, traffic accidents in particular, which might explain their high prevalence following commuting accidents^3^^,^^8^.

Characterizing the epidemiological profile of workers involved in commuting accidents contributes to the identification of high-risk groups, as well as of their possible consequences to the health of workers^11^^,^^23^. It also enables the implementation of specifically targeted strategies for accident prevention and thus to safeguard the health of workers and reduce government spending^9^. In the present study we found that among the men, the highest rate of accidents corresponded to those aged 20 to 29. In Malta et al.’s nationwide study^24^ rates were similar for age ranges 18 to 29 and 30 to 39. This is precisely the age of the economically active population, whence a higher workload exposes individuals to higher odds of becoming involved in commuting accidents^11^^,^^25^.

Our results indicate that males predominated among accident victims, corresponding to 64.2% of the total of incidents in the analyzed period. This finding agrees, albeit in lesser degree, with those reported by Malta et al.^24^, who found that 76.40% of commuting accidents involved men. This situation might be due to the fact that men represent the largest proportion of workers, therefore the odds for commuting accidents to involve males are also higher. Possible reasons for this difference in proportions include discrimination against women in the labor market and the cultural views on women in Brazil, who are held to be responsible for household chores and child care which may hinder their inclusion in the labor market. However, we should observe that this difference decreased in recent years^26^.

Finally, the Labor Reform from 2017 Law no. 13,467/2017, deserves a few words within the context of the present discussion. According to Costa et al.^28^ this law increased the working hours and reduced the minimum time for meal and rest breaks, which together might have negative impacts, such as poorer performance at work, increased number of work accidents and impaired quality of life among workers^28^. Moreover, this law changed the understanding of the commuting time, which independently from the mode of transport used is no longer considered part of the working hours, since workers are not available to the employer^27^.

## CONCLUSION

A direct consequence of the increasing trend of occurrence of commuting accidents detected in the present study is represented by greater negative impacts on the health of Brazilian workers. The macro-mapping we performed evidences the main high-risk groups and injuries associated with incidents and should inspire micro-mapping to achieve a more detailed analysis of accidents, which is necessary for prevention. However, the Labor Reform from 2017 might at least partially hinder this type of studies, since commuting accidents are no longer considered work accidents. These circumstances might reduce the interest of investigators in commuting accidents as a category of work accidents, as well as lead to poorer reporting/recording of incidents.

Commuting accidents have negative impacts especially in developing countries, such as Brazil, as a function of the economic damage to the social security administration and hospital care. Given the intrinsic relationship between commuting accidents and urban vehicle traffic, government actions are extremely necessary, including research, education of the general population and improvement of the road infrastructure, which is still precarious in many parts of the country. Similarly, employers should invest in commuting safety programs, for instance, by hiring vans or buses to transport their employees.
